# Extracellular hemoglobin: the case of a friend turned foe

**DOI:** 10.3389/fphys.2015.00096

**Published:** 2015-04-20

**Authors:** Isaac K. Quaye

**Affiliations:** Department of Biochemistry, University of Namibia School of MedicineWindhoek, Namibia

**Keywords:** hemoglobin, gaseous molecules, intravascular and extravascular hemolysis, inflammation, ROS

## Abstract

Hemoglobin (Hb) is a highly conserved molecule present in all life forms and functionally tied to the complexity of aerobic organisms on earth in utilizing oxygen from the atmosphere and delivering to cells and tissues. This primary function sustains the energy requirements of cells and maintains cellular homeostasis. Decades of intensive research has presented a paradigm shift that shows how the molecule also functions to facilitate smooth oxygen delivery through the cardiovascular system for cellular bioenergetic homeostasis and signaling for cell function and defense. These roles are particularly highlighted in the binding of Hb to gaseous molecules carbon dioxide (CO_2_), nitric oxide (NO) and carbon monoxide (CO), while also serving indirectly or directly as sources of these signaling molecules. The functional activities impacted by Hb outside of bioenergetics homeostasis, include fertilization, signaling functions, modulation of inflammatory responses for defense and cell viability. These activities are efficiently executed while Hb is sequestered safely within the confines of the red blood cell (rbc). Outside of rbc confines, Hb disaggregates and becomes a danger molecule to cell survival. In these perpectives, Hb function is broadly dichotomous, either a friend in its natural environment providing and facilitating the means for cell function or foe when dislocated from its habitat under stress or pathological condition disrupting cell function. The review presents insights into how this dichotomy in function manifests.

## Introduction

Hemoglobin (Hb), a protein that is found in all life forms was discovered by F L Hunefeld in 1840 at Liepzig University (Sheftel et al., 2012). It consists of a prosthetic heme molecule [Fe II coordinated to a tetrapyrole ring (protoporphyrin IX)] joined to globin chains (Schechter, 2008, 2012). Vincenzo Menghini first established that iron in blood is concentrated in the rbc (Busacchi, 1958). Depending on the species or organism Hb functions to transport or store oxygen, offer protection against reactive oxygen species and detoxify nitric oxide (NO), nitrite and peroxides (Nishi et al., 2008; Schechter, 2008; Tiso et al., 2011). In humans and vertebrates, it lies at the cross road of energy transduction from the external environment to the internal milieu of living systems. Its concentration in blood is 150 g/l so that an adult human body contains approximately 1 kg of Hb (Olsson et al., 2012)The functional activities of hemoglobin are essentially dichotomous: (a) to provide a means for binding to oxygen and facilitate its delivery for cellular bioenergetics homeostasis, (b) to be a direct or indirect source of other gaseous molecules, bind to them and in the process moderate red blood cell (rbc) and cellular health, function, defense or damage to the cells. These activities are friendly, salutary and sustaining to cell health and tissue homeostasis when considered vis-à-vis the high concentration of hemoglobin in rbc or outright inimical to cell survival. This review will first present an overview of the general function and reactivity of Hb highlighting aspects that are friendly to cell health and those that are foe like and damaging. The later part will then have a detailed presentation on the synthesis of hemoglobin in humans, its importance for cell function in relation to binding to gaseous molecules and the harm it causes when released outside of its confinement in rbc.

### General Hb function and reactivity

The history of life on earth is closely linked to the evolution of oxygen supply in the atmosphere. This link is central to energy needs of cells and cellular homeostasis in energy transformation (Taylor and Cummins, 2011). The evolution of cyanobacteria with the potential of harnessing energy from the sun with transduction into stored macromolecules in photosynthesis and the release of oxygen, started the process of oxygenation of the atmosphere on the earth (Semenza, 2007). Life forms with increasing complexity including humans have adapted to oxygen usage through Hb in respiration where oxygen serves as the final electron acceptor in the electron transport chain, localized within the mitochondrion (Rich, 2003). While the structure of Hb is optimal to capture oxygen and deliver it to the tissues, it also presents a challenge of auto-oxidation that can modify its structure and lead to generation of reactive oxygen species (ROS) and cellular damage (Reeder et al., 2008). This dichotomy in function makes hemoglobin a double edged sword with one face as a friend and the other a foe under stress.

The primary function of Hb in the capture and release of oxygen is facilitated by heme (with Fe II) which also enables the reversible binding of carbon monoxide (CO) and NO through heme based sensor proteins (Chay and Brillhart, 1974; Girvan and Munro, 2013) while binding of carbon dioxide (CO_2_) is allosterically regulated in a heterotropic interaction with the terminal α-amino groups of Hb through rbc band 3 protein complex and soluble adenylcyclase sensing (sAC) (Bauer and Kurtz, 1977; De Rosa et al., 2008; Buck and Levin, 2011). The binding of hemoglobin to oxygen molecule does not lead to a change in the oxidative state of iron (Rifkind and Nagababu, 2013). However, because at physiological pH oxygen has a higher redox potential than Fe, sometimes hemoglobin undergoes auto-oxidation with oxygen being reduced to the superoxide anion (O^·−^_2_) and generation of metHb/hemin (Fe^3+^) (Bonaventura et al., 2013). This reaction is particularly enhanced under hypoxia (Abugo and Rifkind, 1994). In hemin/metHb, the binding affinity of globin to heme is weak and can lead to loss of heme. This activity is known to affect about 1–3% of Hb under normal physiological conditions (Alayash, 2004). The generation of superoxide and hemin may be beneficial or detrimental, but the release of heme from Hb binding can be damaging when in excess (Aft and Mueller, 1983; Schmitt et al., 1993; Girvan and Munro, 2013). Superoxide being a reactive oxygen species (ROS) is important for signaling function under normal physiology when present in moderate amounts (Reth, 2002; Taniyama and Griendling, 2003). Similarly hemin has a net positive charge, so it can react with nitrites (anions) whether in oxyhemoglobin or deoxyhemoglobin to regenerate hemin and NO (Cosby et al., 2003; Huang et al., 2005). Because hemin is unable to bind oxygen, it does not take part in oxygen transport. NO on the other hand is important for signaling functions, regulation of vascular tone, modulation of the immune response and moderation of endothelial adhesion molecule expression among others (Guzik et al., 2003; Forstermann and Sessa, 2012). So a limited release of heme under normal circumstances is useful. In addition anti-oxidants in the rbc or in circulation are able to offset any potentially harmful degradative products. These include glutathione, ascorbic acid, glutathione peroxidase and peroxiredoxin in the rbc (Ascenzi et al., 2005; Lutz and Bogdanova, 2013). In circulation the major scavengers that limit hemoglobin and heme damage are haptoglobin and hemopexin respectively (Nielsen and Moestrup, 2009; Reeder, 2010; Tolosano et al., 2010; Belcher et al., 2013; Schaer et al., 2014). However, under stress or pathological conditions, the moderating effect of the scavengers is overwhelmed so that a cascade of ROS generated from Hb auto-oxidation is not dampened. In that circumstance NO can react with superoxide to generate peroxynitrite (ONOO^−^) which sets up a cascade of ROS generation that are damaging to cells and tissues (Griendling and FitzGerald, 2003; Pacher et al., 2007). This is augmented by the release of heme intravascularly or extravascularly (Schaer et al., 2013).

Hemoglobin binding to CO and CO_2_ also serves important signaling functions (Maines, 1997; Otterbein et al., 2000; Schechter, 2008). CO has been shown as a signaling molecule in the brain, a messenger molecule in the gastrointestinal tract, and an inducer of hyperpolarization in circular smooth muscle cells (Maines, 1997; Amano and Camara, 2013). CO_2_ which is produced largely in the TCA cycle in cellular metabolism affects tissue homeostasis (e.g., disrupted pH changes) that can lead to death or play an important role in inhibiting inflammation (Buck and Levin, 2011; Cummins et al., 2014). So Hb not only ensures cellular energy supply through oxygen delivery, but also facilitates cellular homeostasis through signaling functions of the gases to which it binds. These functional activities of hemoglobin are friendly, sustaining and salubrious to cell health and tissue homeostasis when considered vis-à-vis the high concentration of hemoglobin in rbc. The review will presently give details on the synthesis of hemoglobin in humans, its importance for cell function and the harm it causes when released outside of its confinement in rbc.

## Human hemoglobin synthesis

Human hemoglobin is produced in erythroid cells and in adults is made up of two major tetramers, HbA (97%) consisting of two alpha and two beta globin polypeptides (α_2_β_2_) and HbA_2_ (2%) consisting of two alpha and two delta globin polypeptides (α_2_δ_2_) (Perutz, 1979). In the fetus, the Hb molecule referred to as HbF consists of two alpha and two gamma chains (α_2_γ_2_). This phenotype can be present in adults up to 1% (Schechter, 2008). After birth, the gamma chains are replaced with beta chains gradually (Perutz, 1979).

**Figure 1:**
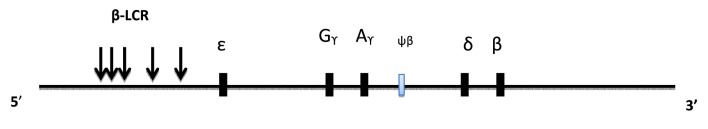
**Organization of the β globin genes on chromosome 11**. Gene expression occurs sequentially from the 5′ region in ontogenesis. The lightly shaded rectangle represent a pseudogene.

The genes for the alpha globin chains are located on chromosome 16 whereas chromosome 11 harbors the genes for the beta globin chains (Huehns et al., 1964). During ontogenesis, the embryonic alpha chains (ζ) are replaced by the adult type (α) which is maintained throughout adulthood (Huehns et al., 1964). However, the expression of the genes for the globin chains in erythroid cells differ based on the developmental stage. The β-globin genes are arranged sequentially from the 5′ region closest to the locus control region (LCR), in the order in which they are expressed during ontogenesis toward the 3′ region (Huehns et al., 1964). This order is ε (HBE), G_γ_(HBG2), A_γ_(HBG1), δ(HBD), and β(HBB)-3′) expressed depending on switches in gene expression (Figure 1) (Huehns et al., 1964). These switches are from embryonic (HBE) to fetal (HBG2 and HBG1) stage and fetal to adult stage (HBD and HBB), regulated by the LCR of the globin gene cluster (Schechter, 2008; Razin et al., 2012; Moleirinho et al., 2013). The HBB gene is more highly expressed in adults than the HBD gene which is why adult hemoglobin is made up largely (97%) of HbA and about 2% of HbA_2_. The prosthetic group heme is synthesized through a series of eight step enzymatic reactions half of which occurs in the cytosol and a half in mitochondria (Heinemann et al., 2008; Chiabrando et al., 2014). After the final step which occurs in the mitochondria matrix, heme is targeted into hemoproteins either membrane bound or in the cytosol (Schultz et al., 2010; Larsen et al., 2012; Korolnek and Hamza, 2014). Hemoglobin and myoglobin acquire heme in the cytosol (Bruns and London, 1965). In these compounds heme (type b) is non-covalently coordinated to each globin chain (alpha and beta) through Fe II. It is postulated that heme acquired into cytosolic globin molecules may be derived from mitochondria or imported through plasma membrane importers or the endocytic pathway (Wu et al., 2006; Korolnek and Hamza, 2014).

## The friendly role of hemoglobin in cell physiology

The friendly role of Hb is defined in part by its ability to bind gases in particular O_2_, NO, CO and CO_2_ for transport. The binding affinity of the gases to Hb through heme are in the order NO>>CO>> O_2_ (Chapman and Cokelet, 1997; Reeder, 2010; Olsson et al., 2012). This activity is fundamental to the survival of multicellular organisms. Indeed the principal function of the cardiovascular system is essentially to deliver oxygen and nutrients, while facilitating the removal of carbon dioxide and metabolic waste in the process (Mairbaurl and Weber, 2012).

### Hemoglobin and O_2_

The transport of oxygen from the lungs to tissues is mediated by the Hb tetramer with heme iron in the ferrous state (Fe^2+^) binding four oxygen molecules (Reedy and Gibney, 2004; Ascenzi et al., 2005; Buehler and Alayash, 2005). In the lungs oxygen diffuses across the alveolar barrier into the blood where it is bound by Hb (Eaton, 1974). Through the activity of the cardiovascular system, oxygen is delivered into tissues by way of the microcirculation comprising the arterioles, venules and capillaries to maintain cellular bioenergetics homeostasis (Haldar and Stamler, 2013). The blood oxygen content which invariably depends on hemoglobin oxygen saturation determines the amount of oxygen delivered to tissues (Mairbaurl and Weber, 2012; Haldar and Stamler, 2013). A decrease in pH or an increase in the partial pressure of CO_2_ (*P CO_2_*) in the systemic capillaries decreases the affinity of oxygen for Hb (Bohr Effect) leading to tissue delivery of oxygen (Dash and Bassingthwaighte, 2006). Tissue oxygen gradient in turn regulates blood flow in the microcirculation, levels of erythropoietin synthesized for erythropoiesis and oxygen delivery (Siri et al., 1966; Hebbel et al., 1978). Hypoxia defined as decreased oxygen levels in the microenvironment of cells or tissues relative to the normal physiological state (demand does not match supply), increases tissue perfusion, erythropoiesis and oxygen delivery (Mairbaurl, 1994; Semenza, 2009, 2011). Hyperoxia has the reverse effect (Piantadosi, 1999b). When oxygen is delivered in the tissues, cytochrome oxidase, the final enzyme in the mitochondria electron transport chain uses the oxygen as the final electron acceptor to generate ATP.

In addition nitric oxide synthase isoforms utilize molecular oxygen together with NADPH and arginine as substrates to generate NO, an important signaling molecule in the central nervous system (CNS) and peripheral nervous system (Robinson et al., 2011). NO can also be generated from a reaction between deoxyhemoglobin and nitrite following oxygen delivery or in hypoxic conditions (Huang et al., 2005).

The microcirculation is facilitated by cellular adaptations to hypoxia which includes improved ventilation and lung perfusion, augmented cardiac contractility, increased vasodilation and regulated ATP release (Jagger et al., 2001; Sprague et al., 2007; Gladwin and Kim-Shapiro, 2012). At the molecular level, a transcription factor, hypoxia inducible factor (HIF) coordinates the responses to hypoxia (Semenza, 2009; Scholz and Taylor, 2013) through the activity of hydroxylases. When oxygen is available, hydroxylases (which are absolutely dependent on molecular oxygen) are activated to hydroxylate HIF for degradation, whereas limited oxygen supply leads to HIF stabilization and expression of genes affecting vascular tone, erythropoiesis, angiogenesis and metabolism (Schofield and Ratcliffe, 2005; Taylor, 2008; Eltzschig and Carmeliet, 2011). These coordinated activities ensure a smooth link for delivery of oxygen and nutrients to tissues with removal of waste and carbon dioxide, so that adequate blood flow through the microcirculation and attendant oxygen supply is maintained for cell health and survival. Decreases in rbc hemoglobin as occurs in hemolysis, or pathological states such as sickle cell disease, malaria, paroxysmal nocturnal hemoglobinuria (PNH), directly leads to decrease in tissue oxygen delivery with attendant pathological consequences (Sikora et al., 2014). The process of oxygen delivery is also regulated by allosteric factors including protons (H^+^), CO_2_, organic phosphates, chloride (Cl-) and 2,3-bisphosphoglycerate (Mairbaurl and Weber, 2012). Structural changes at the molecular level in hemoglobin that alters the affinity of the molecule for oxygen or its response to the allosteric modulators, also contribute to the reversible binding to oxygen. The primary function of Hb in oxygen delivery is clearly also coupled to maintaining energy homeostasis, through processes that augment oxygen availability to cells and tissues.

### Hemoglobin and CO_2_

In contrast to oxygen, carbon dioxide is generated in multicellular organisms through the TCA cycle and during aerobic metabolism (Buck and Levin, 2011; Cummins et al., 2014). Carbon dioxide not only contributes to the buffering system in blood but also engages in signaling activity in critical life processes including sperm activation, blood flow and dampening inflammation (Boatman and Robbins, 1991; Buck and Levin, 2011; Taylor and Cummins, 2011; Cummins et al., 2014). As part of the buffering systems in the blood, the enzyme carbonic anhydrase hydrates CO_2_ to H_2_CO_3_ which is transported to the lungs where it readily dissociates through the activity of carbonic anhydrase to HCO^−^_3_ and H+, with CO_2_ release and exhalation. Any remaining carbon dioxide in tissues is bound by hemoglobin and sent through the circulation to the lungs for exhalation. A decrease in pH or an increase in the partial pressure of oxygen (PO_2_) in the pulmonary capillaries decreases the affinity of CO_2_ for Hb leading to the release of CO_2_ from the blood and exhalation (Haldane effect), (Dash and Bassingthwaighte, 2006).

Elevation in carbon dioxide modulates immune response through inhibition of NF-κB, IL-6, TNF-alpha and mast cell degranulation (Oliver et al., 2012; Cummins et al., 2014). These activities lead to reduced inflammatory response. Carbon dioxide sensing is also known to inhibit innate immune response by altering NF-kB signaling (Helenius et al., 2009; Cummins et al., 2014). One of the major roles of CO_2_ sensing is the regulation of the signaling activity of adenylyl cyclase to generate cAMP (Kamenetsky et al., 2006). The latter function is particularly important as cAMP is generated in microdomains throughout the cell to fine tune intracellular signals through the activity of soluble adenylyl cyclase (sAC) (Oliver et al., 2012). sAC is not dependent on G-protein signaling but rather intracellular signals such as HCO^−^_3_, calcium and ATP (Cummins et al., 2014). In this regard sAC also participates in CO_2_ dependent regulation of ATP generation depending on the amount of CO_2_ released in the metabolic activity of the cell (Buck and Levin, 2011). Two signaling activities of sAC that requires mentioning in this review are the bicarbonate dependent activation of sperm to swim in the female reproductive tract in a process referred to as capacitation and bicarbonate dependent beating of cilia in the alveolar epithelial cells that help in defense against inhaled pathogens or particles (Huckstepp and Dale, 2011). So capacitation for life's beginnings and salutary cellular activities are important functions of carbon dioxide sensing modulated by Hb. If one considers that the partial pressure of CO_2_ regulates ventilation rate and variations in pH and HCO^−^_3_ in the cell can elicit responses such as changes in breathing rate, metabolism, fertility, immune response and gene expression, Hb binding to CO_2_ indirectly modulates these processes that contribute to the foundation of life, its sustenance and maintenance of optimal health through signaling function that protects against pathogens and subdue inflammation.

### Hemoglobin and NO

Nitric oxide (NO) is an important signaling molecule, recognized in the Nobel Prize award in 1998 for Physiology and Medicine (Butler, 2006). It is produced in mammalian cells mainly by enzymatic and non-enzymatic mechanisms. The enzymatic mechanism is through nitric oxide synthase which is found in three different isoforms (nNOS:NOS I, iNOS:NOS II, eNOS) depending on the cell source; n:neurons, i: inducible (macrophages mainly and sometimes hepatocytes), and e: endothelium (Alderton et al., 2001). The main substrate for the enzymes is L-arginine, which is transported into cells by the cationic amino acid transporters (CAT) (Alderton et al., 2001; Hill et al., 2010). The co-substrates are reduced nicotinamide adenine dinucleotide phosphate (NADPH) and oxygen while tetrahydrobiopterin (BH_4_), flavin adenine dinucleotide (FAD) and flavin mononucleotide (FMN) function as cofactors modulated by calmodulin in the redox reaction (Stuehr, 2004; Stuehr et al., 2004). The non-enzymatic source of NO is largely from nitrites (Lundberg and Weitzberg, 2010). Deoxyhemoglobin and deoxymyoglobin by virtue of their nitrite reductase activity (Curtis et al., 2012; Gladwin and Kim-Shapiro, 2012; Kapil et al., 2014; Gao et al., 2015) react with nitrites to generate NO under tissue hypoxia or ischemia. NO acts as a toxin and signaling molecule. The toxic effect is in the generation of ROS (peroxynitrite) from interaction with heme (ferrous iron) or hemin (ferric iron) following Hb auto-oxidation to generate superoxide (Pacher et al., 2007; Beckman, 2009; Hill et al., 2010). This activity functions in the killing of microbes but can also lead to damage of proteins, DNA and lipids derived from peroxynitrite release (Koppenol, 2001). For example NO is toxic to aconitase in the TCA cycle and heme-copper terminal oxidases in the respiratory chain, responsible for peroxynitrites generation, damage to DNA and release of lipid radicals (Nathan and Ding, 2010; Forstermann and Sessa, 2012). This obviously is a problem. Fortunately Hb also functions as nitric oxide dioxygenase (NOD) converting nitric oxide and oxygen to nitrates using NADPH as cofactor so that NO as a ROS source is attenuated (Gardner, 2012). The NOD function of Hb ensures continual energy supply necessary for homeostasis in cellular bioenergetics and reduction in potential damage to cells from NO mediated ROS.

NO signaling is either through soluble guanylate cyclase (sGC) to facilitate vascular homeostasis (blood flow) by modulating vascular tone, inhibiting thrombosis and regulating the expression of endothelial adhesion molecules (Shiva et al., 2011) or through modification of proteins by nitrosylation in metalloproteins, nitrosation or oxidation of protein side chains (Hill et al., 2010; Haldar and Stamler, 2013). It is also important in skeletal muscle contraction, CNS signaling and host resistance to infection. Hb NOD function also means that the role of NO as a signaling molecule can be negatively affected, leading to reduced blood flow. However, NO removal through NOD mediated activity appears to correlate with activation of sGC so that steady state levels are achieved. As a compensatory mechanism, Hb and myoglobin act as a sink for NO releasing it during times of need and generate it from nitrites (Gardner, 2012).

### Hemoglobin and CO

CO is produced in cells by heme oxygenase (HO encoded by HMOX genes) mediated breakdown of heme to give CO, biliverdin and iron (Fe^2+^) (Tenhunen et al., 1969). Two main HO isoforms, HO-1 and HO-2 are known which are induced and constitutive respectively (Tenhunen et al., 1969; Maines et al., 1974; Yoshida and Kikuchi, 1974; Maines, 2000). HO-1 is expressed basally in the liver and spleen while HO-2 is basally and largely expressed in the brain (Immenschuh et al., 2010; Farrugia and Szurszewski, 2014). Other minor sources include lipid peroxidation and progesterone activity (Delivoria-Papadopoulos et al., 1974; Wolff, 1976). Induced HO-1 expression is through a wide variety of stimuli including inflammation, injury, ROS, hypoxia, ischemia and hypothermia (Beckman et al., 2009; Immenschuh et al., 2010; Kacimi et al., 2011). So cells that express HO-2 stably produce CO, while stimuli dependent CO generation from HO-1 expression varies depending on cellular environment (Farrugia and Szurszewski, 2014).

CO is a signaling molecule with antioxidant and anti-inflammatory activities (Farrugia and Szurszewski, 2014). It activates soluble guanylyl cyclase (Ramos et al., 1989; Lutz and Bogdanova, 2013) to generate cyclic GMP (cGMP) for signaling based on the cellular environment (Maines, 2000). In general, the signaling function of CO parallels that of NO (although not as potent) as a neuromessenger and regulator of vascular tone (Furchgott and Jothianandan, 1991). The two gases are involved in a negative feedback regulation with NO activating HO-1 expression, while CO and HO-1 inhibit NO synthesis, (White and Marletta, 1992; Durante et al., 1997; Zuckerbraun et al., 2005). CO is biologically more stable than NO and therefore can exert effects distant from its site of production in addition to local effects (Farrugia and Szurszewski, 2014). The functional activity of CO relates to its high affinity for transition metals. Since iron is the most abundant transition metal in cells, CO competes for binding to various heme proteins, particularly Hb. It binds to hemoglobin with an affinity that is 210–250 times that of molecular oxygen (Haldane, 1895; Nasmith and Graham, 1906; Amano and Camara, 2013). Partial occupation of CO at the oxygen binding site of Hb, inhibits oxygen release from other heme sites (Ryter and Choi, 2013). Hemoglobin can therefore scavenge cellular CO to reduce its physiological impact but at the same time render it incapable of carrying oxygen. The baseline carboxyhemoglobin (CO-Hb) level in man is 0.1–1.0% (Rudra et al., 2010). If this level reaches 10–30%, it may cause headache, dizziness and shortness of breath (Gorman et al., 2003; Sen et al., 2010; La Fauci et al., 2012). Levels above 30% are harmful and can lead to severe headache, vomiting, syncope, cardiac arrhythmias and death (Piantadosi, 1999a; Gorman et al., 2003).

The brain maintains the highest expression of HO, highlighting the importance of CO as a signaling molecule in the brain (Leffler et al., 2011). In the brain, CO dilates cerebral arteries and arterioles by activating smooth muscle large conductance Ca^2+^ activated K^+^ channels or “Big potassium” channel (BK_ca_) (Leffler et al., 2011) by a mechanism that is unrelated to sGC activation (Koneru and Leffler, 2004; Telezhkin et al., 2011). The activation of BK_ca_ channel activity leads to vasodilation promoting brain vascular system health. CO also provides protection for the cerebral vasculature (Parfenova et al., 2005). In hemolysis, when heme availability is augmented, heme binding to the BKca channel deactivates it, which is reversed through CO binding. Therefore, CO generation in hemolysis makes regulation of the CO concentration particularly important during ischemia-reperfusion not only in the brain but also in transplantation (Sata et al., 2001). CO has emerged as a choice therapeutic agent in cerebral malaria (CM). CM is a life threatening neurological complication of *Plasmodium falciparum* infection in children and adults, with the majority of cases (>90%) occurring in children <5 years old in sub-Saharan Africa (Pena et al., 2012; Murray et al., 2014a; Christensen and Eslick, 2015). The incidence rate is 1120/100,000 annually in endemic Africa, with mortality of 13-20% (Idro et al., 2010b; Postels and Birbeck, 2013). Surviving children from the disease endure long term neurological and cognitive deficits (Brewster et al., 1990; Idro et al., 2010a; Postels et al., 2012). Although the pathophysiology is not completely known, current understanding outlines sequestration of infected RBC leading to changes in brain microcirculation, cerebral inflammation, vascular dysfunction and breakdown in blood brain barrier (Shikani et al., 2012; Postels and Birbeck, 2013). Using *P. berghei* ANKA mouse models, HO-1/CO was shown to completely inhibit CM and facilitate the resolution of inflammation in the disease state (Pamplona et al., 2007; Pena et al., 2012). Because of the potential danger of CO binding to heme containing molecules in particular Hb, CO releasing molecules with limited formation of CO-Hb are now being developed (Motterlini et al., 2005; Vera et al., 2005) for CM treatment. CO is also under evaluation for use as a vasodilator and inhibitor of ischemia reperfusion injury, an immunosuppressant in transplantation, (Sato et al., 2001; Otterbein et al., 2003), and as an anticancer agent (Wegiel et al., 2013). Additional roles of CO that are gaining attention include modulation of the innate and adaptive immune response, inflammation (Otterbein et al., 2000), cell proliferation, (Morita et al., 1997) apoptosis (Brouard et al., 2000), mitochondrial biogenesis (Suliman et al., 2007) and autophagy (Lee et al., 2014). Clearly Hb as a source of CO and scavenger of CO confers significant salutary impact on brain health (facilitating nutrient and oxygen delivery, limiting inflammation) and enabling maintenance of healthy cerebral vasculature.

### Hemoglobin as an antioxidant/defense molecule

Hemoglobin in other vertebrates contains a number of thiols that exert antioxidant defenses (Moleirinho et al., 2013). In humans only one reactive cysteine is present at the 93rd position of the β-chain (Vitturi et al., 2013). The Hb β93 cysteine has been observed to moderate heme reactivity leading to auto-oxidation (Balagopalakrishna et al., 1998). It scavenges reactive species (hydrogen peroxide and superoxide anion) generated in the heme pocket of the β-globin chain while inhibiting the activity of nitrosating and alkylating agents (Bonaventura et al., 1999; Vitturi et al., 2013). The mechanism of scavenging of hydrogen peroxide and superoxide anion appears to be the provision of safe transfer of electrons to traditional antioxidant systems in the rbc (Winterbourn and Carrell, 1977). This activity is most effective under high oxygen tension (21%), when the risk of auto-oxidation is high, being lost at 4% oxygen saturation (Benesch and Benesch, 1962). When the total concentration of Hb in circulation is taken into consideration, this will have a significant bearing on cellular defense against hydrogen peroxide reactivity, generation of ROS and hemoglobin auto-oxidation.

### Hb in cellular immune modulation

**Figure 2:**
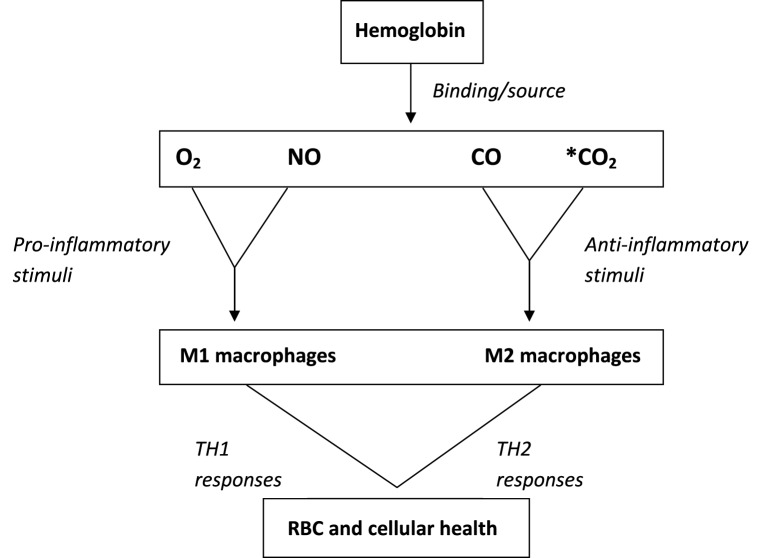
**Hemoglobin functional activities in promoting rbc and cellular health**. (see text for details). ^*^Binding of Hb to CO_2_ is through terminal α-amino groups of Hb in contrast to the other gases.

Central to cellular defense is the host immune response which adapts to confer protection against infection or tissue damage. The cells at the center of this adaptation are the myelomonocytic cell lineage (de Back et al., 2014; Hamilton et al., 2014; Muraille et al., 2014). In the event of an inflammation or infection, the blood monocytes are recruited into tissues where they mature into macrophages or dendritic cells (Geissmann et al., 2003). Macrophages are polarized into M1 and M2 phenotypes depending on the type of stimuli they receive and cytokine profile they generate. In general M1 macrophages are elicited by pro-inflammatory stimuli, while M2 macrophages by anti-inflammatory stimuli (Lawrence and Natoli, 2011; Muraille et al., 2014). In the scheme of activities of hemoglobin modulation of the generation and function of the gaseous molecules NO, CO, and CO_2_, hemoglobin will indirectly influence the M1 and M2 macrophage phenotype and therefore the TH1 and TH2 immune cytokine profiles, (Lee and Chau, 2002; Alcaraz et al., 2003; Murray et al., 2014b). This will have significant implication for cellular defense against damage and pathogens through modulation of TH1 and TH2 responses (Figure 2). This function requires further interrogation.

### Summary: hemoglobin: life's pivotal heart beat

**Figure 3:**
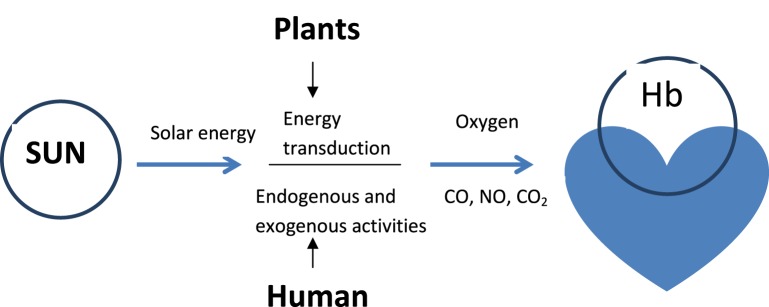
**Hemoglobin: life's core friend in sustaining cardiovascular health**. Hb binding to gaseous molecules through heme (O_2_, CO, NO) or terminal α-amino groups of Hb (CO_2_) facilitates cardiovascular function and health.

In the natural environment hemoglobin is the support base for life, ensuring that vital solar energy captured by plants for photosynthesis with release of oxygen is channeled to humans and vertebrates to sustain the energy requirements of cells and life. It supports life processes by binding to gaseous molecules with major functions of efficient blood flow in the vasculature, oxygen delivery and bioenergetics homeostasis. The cumulative processes for oxygen supply and delivery to tissues is associated with modulation of activities that not only constitute a threat to vascular health but also cellular health in general. These activities which include mitochondria biogenesis, signaling functions, regulation of inflammation, immune responses and cell viability, palpably projects Hb as life's pivotal heart beat (Figure 3) without which evolution of life on earth would have been impossible.

## Hemoglobin mediated tissue injury: the foe

The damaging role of Hb under stress or pathological conditions fall under four categories:

- Hemoglobin auto-oxidation within the rbc
- Hemoglobin release outside of its confines in rbc to act as ROS source in the vasculature and tissues
- Hemoglobin scavenging of NO
- Heme release from Hb to augment free radical generation

For reviews covering these topics the reader is referred to Olsson et al. (2012), Schaer et al. (2014), Rother et al. (2005), Larsen et al. (2012), Gladwin and Sachdev (2012) and Tolosano et al. (2010). So, how does Hb that provides such a strong support base for life turn into an enemy?

### Hb auto-oxidation

In general oxygen reversibly binds to Hb without any change in the redox state of iron (Bonaventura et al., 2013). However, at physiological pH, O_2_ has a higher redox potential than aqueous iron (Fe^3+^/Fe^2+^) so oxygen coordinated to heme in hemoglobin accepts an electron from Fe^2+^ heme to form superoxide (O^−^_2_) with oxidation of Fe^2+^ to Fe^3+^ generating met-hemoglobin (hemin) in a stable complex (HbFe^3+^)O^−^_2_, (Weiss, 1964; Faivre et al., 1998; Gardner, 2012). This process is Hb auto-oxidation which is enhanced at acidic pH (low oxygen tension) but reduced at high oxygen tension (Faivre et al., 1998; Aranda et al., 2009; Bonaventura et al., 2013). Within the confines of the rbc, antioxidants enable reduction of Ferric ion to the Fe^2+^ state (Antonini, 1974; Edsall, 1986) so that Fe^3+^ is depleted to cause no damage. Outside of the rbc auto-oxidation is high posing significant danger.

### Injury associated with Hb auto-oxidation and release outside of the rbc

Outside of the rbc, the major antioxidants are haptoglobin for Hb (Schaer et al., 2014) and hemopexin, albumin, and α-1 microglobulin (Fasano et al., 2007; Nordberg et al., 2007; Tolosano et al., 2010) for heme. When hemolysis is severe the capacity of the antioxidants to scavenge is overwhelmed, so metHb and superoxide persist from Hb oxidation to set a cascade of free radical generation to be described presently. First released hemoglobin is closer to the vascular endothelium and can also extravasate into tissues as dimers so that ROS generated in auto-oxidation readily access macromolecules and cause damages (Nathan and Cunningham-Bussel, 2013; Dutra et al., 2014). In that scenario tissue NADPH oxidases and vascular NADPH oxidases facilitate the generation of hydrogen peroxide from superoxide (Klebanoff, 2005). Superoxide also undergoes dismutation by dismutase (superoxide dismutase) to generate hydrogen peroxide which can further oxidize oxyHb/Met-Hb to the ferryl (Fe^4+^ = O^2−^) form and in the process produce hydroxyl radical (OH^−^) through Fenton chemistry (O^•−^_2_ + H_2_O_2_ → O_2_ + OH^−^ + OH^−^), (Brawn and Fridovich, 1980; Klebanoff et al., 1989; Pastor et al., 2000; Liochev and Fridovich, 2002). These are highly reactive species that react with globin chains and porphyrin rings to form globin and porphyrin ring radicals (Alayash, 2011), DNA to form adducts and lipids to form peroxides (Cadet et al., 1999; Marnett et al., 2003). The oxidized hemoglobin species may resist scavenging and sustain tissue injury, particularly the vascular endothelium (Goldman et al., 1998; Rother et al., 2005). Moreover when superoxide is formed in large quantities, because NO has a free electron in its outer shell it can react with the superoxide to form peroxynitrite radical (ONOO^−^) which has a high reactivity with heme or hemin and cellular macromolecules (Brawn and Fridovich, 1980; Halliwell, 1996; Burney et al., 1999; Marnett et al., 2003). Hydrogen peroxide can also cause intermolecular cross linking of tyrosine residues in the globin chains which are reported to contribute to atherosclerotic plaques in cardiovascular disease (Doyle et al., 1985; Halliwell et al., 1999). So severe intravascular and extravascular hemolysis lead to the damage of the vasculature as are exposed cells and tissues at the cell membrane, mitochondria and cytoskeleton, a very unfriendly, foe type activity arising from Hb.

### Hb and NO scavenging

Hemoglobin reacts rapidly and irreversibly with nitric oxide (10^7^ m^−1^ s^−1^) to generate metHb and nitrate. The rate of this reaction is such that a very small quantity of Hb can cause significant NO depletion (Olson et al., 2004). The significance of this activity was demonstrated in the reversal of acetylcholine mediated aortic arc relaxation in rabbits by cell free Hb at concentrations >1 mg/ml (Nakai et al., 1996). NO is an important signaling molecule in maintaining vascular tone and blood pressure, angiogenesis, platelet function, vascular smooth muscle cell proliferation and inflammation (Shaul, 2002; Sessa, 2004; Atochin and Huang, 2011) as alluded to previously. By scavenging NO, cell free Hb significantly impair vascular function, bioenergetics homeostasis and portend significant morbidity.

### Heme release from cell free Hb

Oxidized and denatured hemoglobins lose their attached heme readily leading to heme mediated ROS generation (Balla et al., 1991a,b). Heme mediated ROS generation is largely attributed to the redox function of iron, which mediates reductive or oxidative reactions involving the transfer or acceptance of electrons (Dutra et al., 2014). This redox function makes Hb also function as a major inducer of ROS because of its large concentration in the rbc (Jeney et al., 2013). Heme is lipophilic and can intercalate into cell membranes to disrupt membrane integrity (Schmitt et al., 1993). Other downstream processes follow similar path to that outlined for Hb auto oxidation which involves generation of free radicals (Huertas and Palange, 2011; Dutra and Bozza, 2014) pro-inflammatory activities and cytotoxicity (Larsen et al., 2012). The scavenger proteins haptoglobin (Hp) and hemopexin (Hpx) will normally scavenge cell free Hb and heme respectively obviating any ROS related activity if not in excess (Hvidberg et al., 2005; Quaye, 2008; Tolosano et al., 2010; Schaer et al., 2014). Hb bound haptoglobin is targeted for elimination through the monocyte/macrophage scavenger receptor CD163 (Schaer et al., 2014) while is heme first bound by albumin, transferred to Hpx and the heme-Hpx complex targeted for clearance by the LDL receptor related protein 1 (LRP 1/CD91 in hepatocytes in particular (Ascenzi and Fasano, 2007; Schaer et al., 2014). In addition heme can also be scavenged by α-1 microglobulin (Allhorn et al., 2002) and high density lipoproteins (Miller and Gladwin, 2012). When levels of these proteins fall due to excessive hemolysis then cellular/tissue damage is cascaded. For further reading the reader is referred to Schaer et al. (2014), Tolosano et al. (2010), Alayash (2011), Kristiansen et al. (2001), Nielsen and Moestrup (2009), Hrkal and Klementova (1984), Balla et al. (1991b), Hvidberg et al. (2005), Dutra and Bozza (2014) and Dutra et al. (2014).

### Mechanism of Hb release

Red blood cells lack a nucleus or mitochondria so injury mainly affects the cell membrane leading to cell rupture and release of hemoglobin either extra-vascularly or intra-vascularly (Balla et al., 1991b). In severe insults (infection, complement fixation and activation, trauma) where the rbc membrane is compromised, hemoglobin is released outside of its confines in the rbc posing danger not only to the vasculature but also to exposed tissues following extravasation (Jeney et al., 2013; Dutra et al., 2014; Schaer et al., 2014).

#### Extravascular and intravascular hemolysis

Human red blood cells have a normal life span of 120 days (Dhaliwal et al., 2004; Sugawara et al., 2010). If this life span is curtailed either through breakdown of the cell within the blood vessel or outside of the vessels leading to the release of hemoglobin, the process is referred to as intravascular or extravascular hemolysis respectively (Dhaliwal et al., 2004; Sugawara et al., 2010; Mairbaurl, 2013). Normally when hemoglobin reaches a life span of 120 days, it is removed by the spleen, bone marrow macrophage phagocytosis and liver Kupffer cells. Part of the trigger for removal is the presence of Heinz bodies which are aggregates of oxidized or denatured hemoglobin in the rbc, Howell-jolly bodies (inclusions of nuclear chromatin), siderocytes (rbcs with granules of iron that is not part of Hb) and Pappenheimer bodies (inclusion bodies formed by phagosomes ingesting large amounts of iron) (Winslow et al., 1978; Weatherall, 1995, 2011; Sugawara et al., 2010). The process of rbc removal by the mononuclear phagocyte system in the spleen in particular, does not lead to lysis so endogenous antioxidants including glutathione, ascorbic acid, catalase, glutathione peroxidase and peroxiredoxin-2 in the rbc limit the release of hemoglobin extravascularly (Crespo et al., 1987; Mohandas and Gallagher, 2008). Indeed some hemoglobin may leak and trigger the release of reactive oxygen species but this is limited with respect to severity as the hemoglobin scavenger receptor CD163, and heme scavenger receptor CD91 effectively scavenge the released hemoglobin through haptoglobin and hemopexin respectively (Kristiansen et al., 2001; Hvidberg et al., 2005; Tolosano et al., 2010; Schaer et al., 2013, 2014). Hb can also be released extravascularly from internal bleedings (trauma) due to damaged micro- and macro blood vessels.

In intravascular hemolysis, rbcs break up during circulation from oxidative stress, aging or infection releasing Hb directly into the vasculature and subsequently heme (Rother et al., 2005; Jeney et al., 2013; Schaer et al., 2014). Intravascular and extravascular hemolysis serve as potent sources of free radicals that define the hazardous nature of Hb. The amount of Hb released depends on the degree of severity of the oxidative stress or pathological condition which overwhelms natural antioxidants and scavengers in the rbc and plasma.

### Summary: hemoglobin as a danger molecule outside of its confines

In severe extravascular and intravascular hemolysis Hb assumes a foe like nature serving to propagate ROS generation, disrupt cell membranes and cell function, the very activities that it supports when confined. Fortunately, this foe like activities present under abnormal conditions requiring external intervention. The nature and type of the interventions present a challenge but also a major focus of research the world over.

## Conclusion

Hemoglobin is a support base for all life processes physiologically. In the normal, physiological conditions it is salutary, under stress (infection or trauma) the nature that promotes cell health is disrupted leading to real threat and danger to cell function and survival.

### Conflict of interest statement

The author declares that the research was conducted in the absence of any commercial or financial relationships that could be construed as a potential conflict of interest.
